# Evaluation of kidney oxygenation monitoring with near infrared spectroscopy in preterm neonates: kidney location, depth, and laterality differences

**DOI:** 10.1038/s41372-025-02358-2

**Published:** 2025-07-19

**Authors:** Matthew W. Harer, Kate Walker, Lauren Gadek, Shayla Schwingle, Cassandra Nelson, Meg Baker, Elena Alfaro, Adam S. Bauer, Paige Condit

**Affiliations:** 1 Division of Neonatology, Department of Pediatrics, University of Wisconsin School of Medicine and Public Health, Madison, WI USA; 2 University of Wisconsin-Madison, Madison, WI USA; 3 Northwestern University Feinberg School of Medicine, Chicago, IL USA

**Keywords:** Translational research, Paediatrics

## Abstract

**Introduction:**

Near infrared spectroscopy (NIRS) monitoring of preterm kidney oxygenation is increasing. We sought to evaluate kidney positioning and oxygenation differences between the right and left kidneys.

**Methods:**

Preterm neonates <32 weeks’ gestational age were enrolled in a prospective observational NIRS study. Two three-hour epochs of simultaneous bilateral kidney monitoring were performed (5–8 days, 9–14 days). Point of care kidney ultrasound was performed once between days 5 and 12.

**Results:**

There were no right-left differences in skin to superior kidney depth (0.39 and 0.38 cm; *p* = 0.62) or spine to kidney distance (1.06 and 1.02 cm; *p* = 0.31). There was a difference in right-left skin to inferior kidney depth (2.15 and 2.04 cm; *p* = 0.02). There was higher oxygenation in the left kidney compared to the right (65.6 vs. 62.7%, *p* < 0.01). Hourly individual differences of >10% were seen in 37% of neonates.

**Conclusions:**

Differences in kidney depth and right-left oxygenation exist in preterm neonates.

## Introduction

Critically ill preterm neonates frequently experience acute kidney injury (AKI) in the neonatal intensive care unit (NICU), which independently increases their risk of death and long-term complications, including chronic kidney disease (CKD) [1–5]. Despite the significant short- and long-term detrimental effects of AKI, there are currently no approved neonatal AKI therapies, largely because there is not a diagnostic method to effectively detect AKI prior to or at the time of injury [6]. One method to identify kidney stress or early injury prior to permanent damage, without reliance on blood sampling or urine collection, is using non-invasive near-infrared spectroscopy (NIRS) monitoring of kidney oxygenation [7]. NIRS is a continuous monitoring tool already frequently used in the NICU and has been shown to be safe in the neonatal population [8]. NIRS monitoring to detect changes in kidney oxygenation in both preterm and term neonates is being used with increasing frequency, and baseline values in the first two weeks of life have been established [9, 10].

Understanding the anatomic location of the kidneys in neonates is essential to effectively use NIRS to measure kidney oxygenation in preterm neonates. Typically, the right kidney is lower in the abdomen, shifted medially, and is slightly smaller when compared to the left kidney, due to displacement from the liver [11]. Because of this anatomical asymmetry, it is possible the right and left kidneys may have different regional tissue oxygenation. In previous cerebral NIRS studies, simultaneous tissue oxygenation of the right and left cerebral hemispheres has differed by up to ten percentage points, suggesting that kidney oxygenation may also have laterality differences [12]. To measure kidney oxygenation, NIRS sensors are typically placed on either the right or left flank horizontally between T12 and L2 [13]. In neonatal studies, this placement has not yet been confirmed by ultrasound imaging. Similarly, the depth of the kidney below the skin is not known in preterm neonates of different sizes and gestational ages. This is important to consider as the near infrared light for the Medtronic INVOS machines with the neonatal sensor, and the shallow sensor at 30 mm and deep sensor at 40 mm, are reported to make an arc ~1.5–2 cm deep [14, 15]. Establishing right-left kidney oxygenation differences and kidney depth is critical for developing accurate monitoring and treatment guidelines for the preterm population.

The aim of our study was to evaluate kidney oxygenation monitoring with NIRS in preterm neonates born less than 32 weeks’ gestation. We utilized a two-pronged approach – evaluating simultaneous laterality differences in kidney oxygenation between the right and left kidney and by determining the depth of the kidney below the skin on the flank. We hypothesized there would not be a clinically significant difference in the averages of simultaneous kidney oxygenation values of the right and left kidney and that the inferior aspect of some kidneys would be less than two cm below the skin.

## Methods

This prospective observational study was performed at the level III NICU at UnityPoint Health-Meriter Hospital (Madison, WI, USA) between 2021 and 2024. The study protocol and procedures were reviewed and approved by the UW-Madison Health Sciences Institutional Review Board. The study team implemented procedures in accordance with the ethical standards of the relevant human subjects’ research oversight committees (institutional, local, and national), including obtaining informed consent from each participant’s legal guardian prior to enrollment. The sample size for the study was based on a primary outcome of difference in kidney oxygenation between participants with and without AKI (which will be reported in a subsequent publication) while the outcomes for this manuscript were secondary outcomes. No randomization procedures or blinding were utilized for this study. This observational study was not registered with clinicaltrials.gov.

### Participants

Neonates born less than 32 weeks gestational age (GA) were approached for informed parental consent. Exclusion of participants occurred if any of the following criteria was met: 1) enrollment and placement of NIRS sensors not possible by 96 h of life, 2) non-English or non-Spanish speaking families, 3) refusal by attending physician, 4) Mothers unavailability to participate in the consent process, 5) investigative team unavailable to consent parent, 6) NIRS monitors unavailable, 7) Documented congenital abnormality of the kidney or urinary tract (Defined as a major anomaly like posterior urethral valves, cystic kidney disease, kidney dysplasia but excluding fetal pyelectasis).

### NIRS monitoring

Kidney oxygenation was measured on the right and left flank using INVOS^TM^ 5100C (Somanetics, Troy, MI, USA) NIRS monitors. These monitors provide only one NIRS parameter, regional oxygenation as a percent (Oxyhemoglobin/(Oxyhemoglobin + Deoxyhemoglobin) *100). The NIRS sensor was placed over a Mepitel adhesive dressing to protect the neonate’s skin per unit protocol for neonates born <34 weeks gestation. Bilateral kidney tissue oxygenation was recorded in two epochs for 2–6 h between the fifth and eighth days of age and again between the ninth and fourteenth days of age. Bilateral monitoring was completed by placing NIRS sensors on both the left and right flank simultaneously. Handling and positioning of neonates was not restricted, and neonates were re-positioned every 3–6 h per unit guidelines.

### Ultrasound imaging

Between five to twelve days of age a point of care kidney ultrasound was conducted by the PI of the study. Using a Sonosite SII (FUJIFILM Sonosite, Bothell, WA, USA) ultrasound machine, a linear probe was placed at T12-L2 underneath the bottom rib and above the iliac crest. Measurements of skin to superior kidney, skin to inferior kidney, and spine to kidney were performed. The GA, birthweight, and birth length were recorded and analyzed with the ultrasound measurements.

### Standard clinical care

The clinical management of each participant was decided by the attending physician. Within the first 24 h of age, all preterm neonates have a basic metabolic profile obtained which includes serum creatinine (sCr) to monitor for imbalance of electrolytes and kidney function. This is repeated at 48–72 h of life and again at one week of age. In cases of a preterm neonate being found at risk of AKI between days two and seven, more frequent sCr monitoring occurs. Following the first seven days of age, the attending physician determines if there is a need for additional sCr and liver function tests. The neonate’s diapers are weighed at three or six-hour intervals during nursing care sessions to determine urine output. Urine output monitoring is changed to ‘diaper counts’ when off intravenous fluids or diuretics.

### Outcome measures

The primary outcome measure of this study was the difference in simultaneous kidney oxygenation between the right and left kidneys. Secondary outcome measures included comparison of simultaneous right and left kidney oxygenation over the course of two separate epochs by GA and by sex, as well as comparison of ultrasound measurements to GA, birthweight, and birth length.

### Statistical analysis

For all continuous variables, the median and interquartile range were calculated. For categorical variables, the number and percent were calculated. The non-parametric Wilcoxon matched pairs signed rank test was used to determine differences in continuous variables for simultaneous oxygenation monitoring. The Mann–Whitney test was used to compare right-left ultrasound measurements and Spearman r correlation was used to compare ultrasound measurements to birth weight, length, and GA. Differences in percent of participants with kidney tissue between 1.5–2 cm on the right and left were compared using a Fisher’s exact test. Statistical analyses were completed using GraphPad Prism, version 9 (GraphPad Software, Boston, MA, USA). A *p*-value < 0.05 was considered statistically significant.

## Results

### Right-left kidney oxygenation

#### Study participants and demographics

**Fig. 1:**
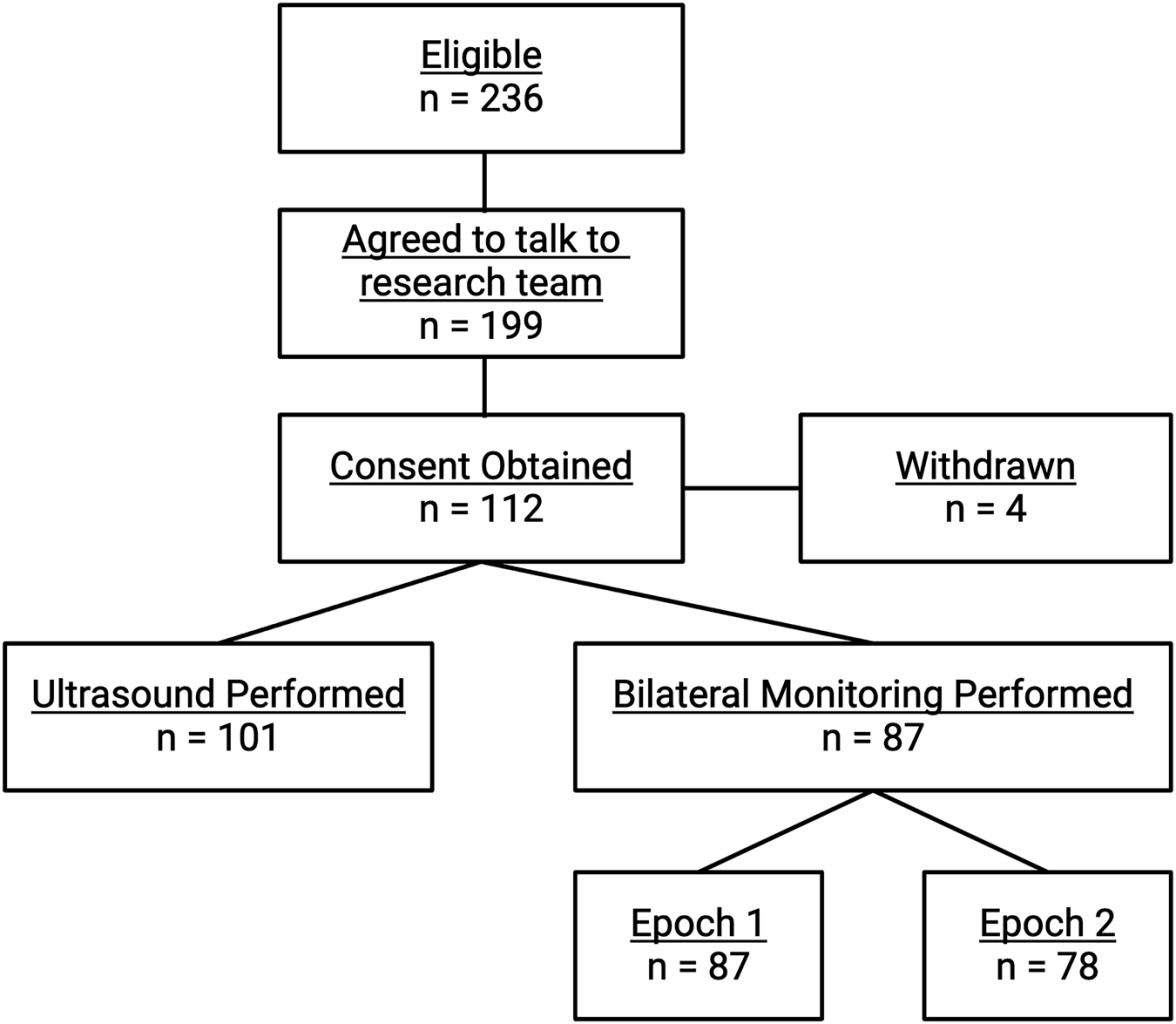
CONSORT diagram. The CONSORT diagram displays the number of eligible patients for the study and subsequently the number that were ultimately included for both the ultrasound analysis and the bilateral monitoring analysis.

A total of 112 neonates enrolled in the study. Demographics of these 112 neonates are described in Table 1. Of these 112 patients, 95 underwent bilateral kidney monitoring during the first time frame, and 70 of these 95 neonates underwent a second period of bilateral monitoring (Fig. 1, Consort Diagram). On average, the neonates underwent their first session of bilateral monitoring on day six and underwent their second session on day eleven.

**Table 1:** Demographics.

	Bilateral monitoring (*n* = 95)	Ultrasound (*n* = 101)
Gestational age (weeks)	29 (27,30)	29 (27,30)
Birth weight (grams)	1215 (943,1450)	1260 (1030,1450)
Sex (male)	55 (57.9%)	58 (57.4%)
Small for gestational age	6 (6.3%)	5 (4.9%)
Ethnicity		
Not Hispanic or Latino	76 (88.4%)	91 (90.1%)
Hispanic or Latino	10 (11.6%)	10 (9.9%)
Race		
White	74 (86.0%)	87 (86.1%)
Black or African American	9 (10.5%)	9 (8.9%)
Asian	1 (1.2%)	2 (2.0%)
Other race	2 (2.3%)	3 (3.0%)

#### Simultaneous bilateral kidney oxygenation monitoring

Analysis of the combined monitoring periods revealed higher oxygenation in the left kidney compared to the right (65.57% (IQR 55.87–74.56) vs. 62.0% (IQR 50.42–72.10), *p* < 0.01). Analysis by epoch also shows higher oxygenation in the left kidney (Epoch 1: 61.80% (IQR 51.87–71.10) vs. 57.88% (IQR 46.98–71.95), *p* < 0.01; Epoch 2: 68.96% (IQR 59.88–76.45) vs. 65.96% (IQR 56.79–73.37), *p* < 0.01).

**Fig. 2:**
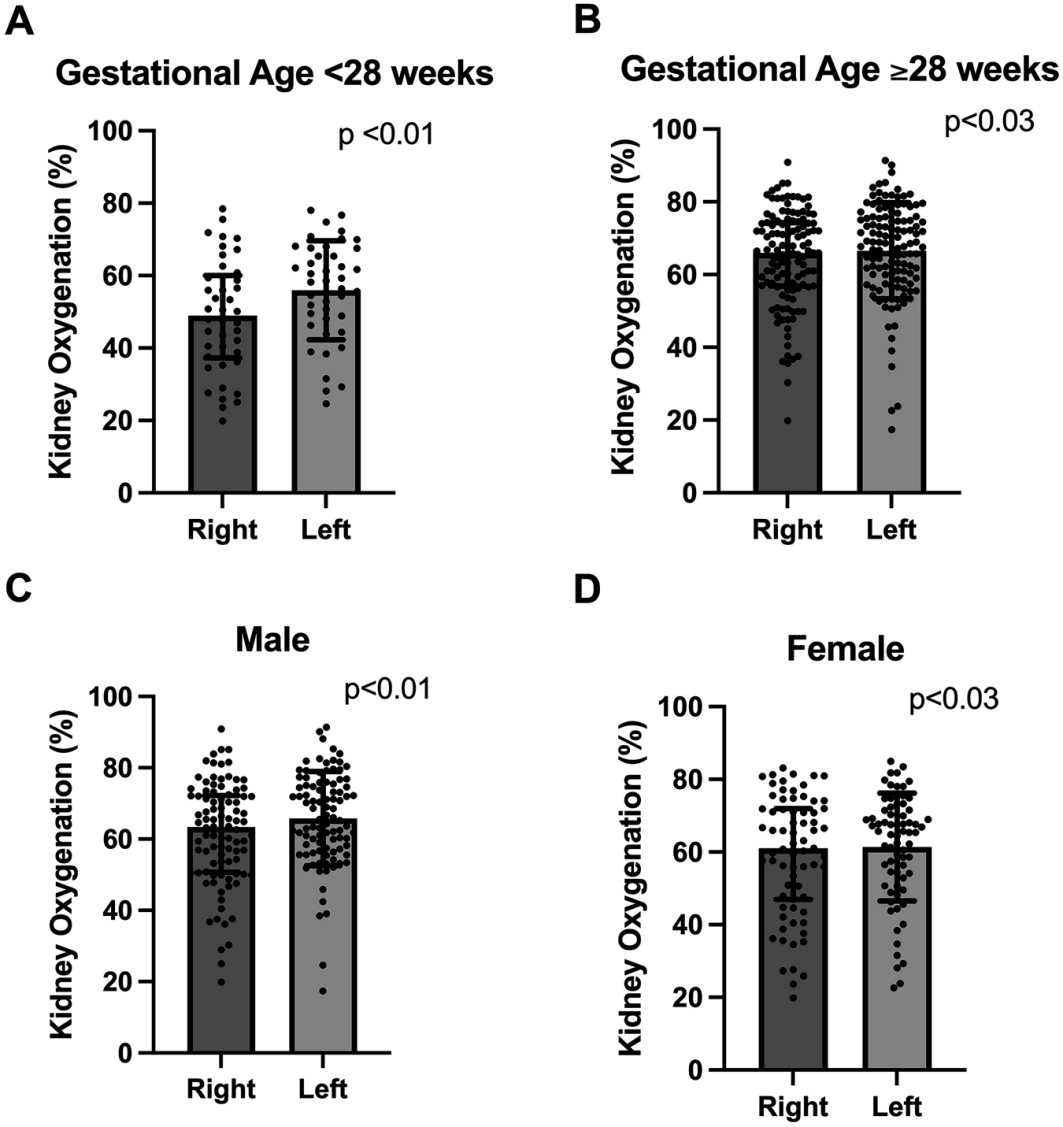
Gestational age and sex differences in right-left kidney oxygenation. This panel of figures demonstrates differences in right-left kidney oxygenation by gestational age and by sex (**A** - Gestational age <28 weeks; **B** - Gestational age ≥28 weeks; **C** - Male; **D** - Female). There are statistically significant differences between the right and left kidney in each of the analyses, with the right always being lower than the left (Wilcoxon matched pairs signed rank test).

Analysis by GA showed higher kidney oxygenation on the left for both <28 weeks GA and 28–31 6/7 weeks GA (<28 weeks: 58.36% vs. 48.97%, *p* < 0.01; 28–31 6/7 weeks: 68.52 vs. 65.96, *p* < 0.01; Fig. 2). Analysis by sex showed higher kidney oxygenation on the left for both males and females (males: 65.82% vs. 63.34%, *p* < 0.01; females: 65.41% vs. 61.03%, *p* = 0.03; Fig. 2).

Evaluation of simultaneous differences by hourly average showed that 63% of neonates had less than a 10-point difference, 27.3% had between a 10–20-point difference, and 9.7% had a greater than 20-point difference between their right and left kidneys. These point differences are displayed as the number of neonates in each category, followed by the percentages in Table 2.

**Table 2:** Point differences in right left kidney oxygenation.

	<10 point difference	10–20 point difference	>20 point difference
All data	104 (63.0%)	45 (27.3%)	16 (9.7%)
Monitoring on days 5–8	62 (71.3%)	17 (19.5%)	8 (9.2%)
Monitoring on days 9–14	44 (56.4%)	26 (33.3%)	8 (10.3%)
Gestational age < 28 weeks	26 (61.9%)	12 (28.6%)	4 (9.5%)
Gestational age ≥ 28 weeks	83 (67.5%)	30 (24.4%)	10 (8.1%)
Male	63 (67.0%)	21 (22.3%)	10 (10.6%)
Female	44 (62.0%)	22 (31.0%)	5 (7.0%)

### Point of care kidney ultrasound

#### Study participants and demographics

Of 112 neonates enrolled, 101 underwent ultrasounds to measure skin to superior kidney, skin to inferior kidney, and spine to kidney distances. Ultrasound was performed at a median of nine days of age. Eleven neonates were unable to have an ultrasound performed due to clinical instability or removal from the study. Demographics are displayed in Table 1.

#### Ultrasound measurements

Analysis of the three measurements revealed no significant differences in median right and left measurements of skin to superior kidney depth (0.39 cm (IQR 0.34–0.43) vs. 0.38 cm (IQR 0.32–0.43); *p* = 0.71) or spine to kidney distance (1.06 cm (IQR 0.94–1.16) vs. 1.02 cm (IQR 0.87–1.16); *p* = 0.33), but there was a difference in right-left skin to inferior kidney depth (2.15 cm (IQR 1.95–2.29) vs. 2.04 cm (IQR 1.86–2.27); *p* = 0.02).

Correlations of skin to superior kidney depth, spine to kidney distance, and skin to inferior kidney depth to birth weight, birth length, and GA are displayed in Table 3. The strongest correlations were to birth weight and birth length, but GA also had significant correlation for all measures except right kidney to spine length.

**Table 3:** Ultrasound correlation.

	Correlation (r)		
	Birth length	Birth weight	Gestational age
Skin to superior kidney (cm)			
Right	0.44**	0.44**	0.34**
Left	0.59**	0.56**	0.52**
Skin to inferior kidney (cm)			
Right	0.75**	0.75**	0.64**
Left	0.66**	0.63**	0.55**
Kidney to spine (cm)			
Right	0.24	0.25*	0.15
Left	0.40**	0.41**	0.25*

*p < 0.05; **p < 0.01.

When comparing right and left kidneys, 26 of 101 (25%) participants did not have right kidney tissue between 1.5 and 2 cm while 42 of 101 (41%) did not have left kidney tissue between 1.5 and 2 cm (*p* = 0.03).

## Discussion

In this prospective study, we sought to evaluate kidney oxygenation monitoring with NIRS in preterm neonates by verifying kidney location and evaluating simultaneous laterality differences in kidney oxygenation. Verifying NIRS monitoring as an effective tool to measure kidney oxygenation is crucial to develop monitoring and treatment guidelines in the future. In this study our main finding is that the left kidney has higher oxygenation than the right kidney by 3.5 percentage points on average. We argue however, that although this difference is statistically significant, it is unlikely to be clinically significant, unless using a specific cut-off for treatment instead of an individual trend. Our point of care ultrasound evaluation of the depth of the kidney in preterm neonates of various gestational ages and weights reveals that the kidney is located immediately under the thin preterm skin, on average only 0.4 cm from the surface of the skin allowing for easy detection of kidney oxygenation. However, in <32-week gestational age preterm neonates, kidney tissue is located between 1.5–2 cm deep 75% of the time on the right, and 59% of the time on the left - so if sensor light travels over 2 cm deep, there may be some detection of ‘peri-kidney’ oxygenation in a minority of patients. Both findings are important contributions towards validating the use of kidney oxygenation monitoring in preterm neonates with NIRS and need to be considered when utilizing NIRS monitoring in the NICU.

In aggregate, our study shows there is a difference in oxygenation between the right and left kidney. This is similar to previously reported differences in cerebral oxygenation between the right and left hemispheres [12]. There are anatomical explanations for finding differential oxygenation – the kidneys are not located in the exact same location on each side of the body and the aorta typically travels along the left side of the spine – perhaps providing higher oxygenation and perfusion to the left kidney [16]. Additionally, the left renal artery is typically shorter and arises more cephalad compared to the right main renal artery, likely due to aorta and kidney positions [17]. When evaluating individual differences, our findings also suggest differential oxygenation between the left and right kidneys. In fact, 37% of neonates had a 1-h average that was >10 percentage points different. This was most pronounced during the second monitoring epoch, when 46% of neonates had a 1-hour average that was >10 percentage points different. This indicates that individual neonates may have a significant differential between right and left kidney oxygenation – so if considering a work-up or treatment of low kidney oxygenation, measurement of the opposite kidney oxygenation is an important first step. For example, within our study, there were twenty-six neonates who had one kidney below 50% and one above at the time of bilateral monitoring. If both kidneys are not checked for oxygenation in these cases and a treatment cut off of 50% is chosen, these neonates may not receive treatment as quickly and efficiently as they need or they could receive treatment that is not necessary.

Potential explanations for why we found a significant differential in right-left kidney oxygenation may exist in our point of care ultrasound findings. When considering depth of sensor light, it is important to consider both the skin to superior kidney distance as well as the skin to inferior distance. The skin to superior kidney distance was found on average to only be 0.4 cm, suggesting that kidney oxygenation should be easily detected and not likely a problem even if preterm neonates develop a small amount of skin edema. This contrasts with adult and pediatric studies that have found that obesity and edema may significantly limit the ability of NIRS to detect kidney oxygenation [18]. Regarding skin to inferior kidney depth, our findings suggest that the deeper arc of light, that may go up to 2 cm deep, could be detecting oxygenation of non-kidney tissues in 25% and 41% off patients depending on the side. On the right side, this could be either liver or large intestine, while on the left side this could be the spleen, colon, or pancreas. There is a possibility that with monitoring kidney oxygenation that the depth of light is not exactly 2 cm, because the testing done on depth of penetration was for cerebral oxygenation, but other testing on various tissues suggests that scattering of light was similar even when not going through the skull [14, 15]. Given the up to 41% of neonates in our study had a kidney depth of less than 2 cm, future development of sensors with either adjustable depth of arcs or a specific preterm sensor with a maximum arc of less than 2 cm may improve the consistency of kidney oxygenation detection. Future studies will need to assess if changes in depth of monitoring results in improved sensitivity or specificity detecting kidney stress or AKI in this population.

Within this study, analysis by GA category (< or >28 weeks) showed statistically significant differences between left and right kidney oxygenation. However, the neonates born before 28-weeks’ gestation had overall lower kidney oxygenation with a median of 58% and 49% for the left and right kidneys, as compared to the neonates born at or after 28-weeks gestation, who had medians of 68.5% and 66%. This trend is similar to previous findings of our research group showing those with lower GA have lower kidney oxygenation in the first week of age [19]. The etiology of this lower kidney oxygenation may be secondary to decreased blood supply to the kidney in the lowest GA neonates or due to higher extraction of oxygen by the kidney due to decreased nephron endowment. With regards to trend in kidney oxygenation over time, we found that kidney oxygenation increased from the first week of age to the second week of age. Kidney oxygenation between days five to eight were 62% and 58% for the left and right kidney while for days nine to 14 it was 69% and 66% respectively. Previous studies have not evaluated how kidney oxygenation changes beyond seven days of age. This pattern suggests that there is a slight increase in kidney oxygenation in the second week of life compared to the first week – perhaps due to improved kidney blood flow or decreased oxygen consumption demands in the kidney. Further research is necessary to evaluate how kidney oxygenation changes throughout all post-menstrual ages in the NICU.

The advantage of our study was that it was prospective, and we enrolled a large number of preterm neonates, but it did have a few important limitations. First, not all infants were able to undergo both epochs of bilateral monitoring due to either transfer to another institution or medical instability. Second, although an ultrasound was performed to assess kidney location, each time a sensor was placed or moved by the nursing staff, an ultrasound was not performed to ensure proper location so we cannot guarantee that during simultaneous bilateral monitoring the sensors were in the exact appropriate position. We also did not have a second member of the team perform ultrasound measurements (to minimize unnecessary interruptions to participants) so cannot perform an interobserver reliability test. Third, since most measurements were during daytime hours, we may have missed any effect of the circadian rhythm on kidney oxygenation. Additionally, we do not have detailed information on nephrotoxins and other factors that may increase the risk of AKI and affect kidney oxygenation. Similarly, we lack simultaneous measurement of hemoglobin saturation to document that there was no desaturation at the time of NIRS measurement. Finally, this is a single center study with significant demographic homogeneity that may not translate to other populations.

In conclusion, our findings suggest there may be differential oxygenation between the right and left kidney and that current neonatal sensors may detect more than just kidney oxygenation in the smallest most preterm neonates. We found crucial oxygenation differences of 10% or greater between the right and left kidneys in 37.0% of the neonates monitored in this study. As a result, when determining future treatment protocols for abnormal kidney oxygenation, it is important to include checking the oxygenation of both kidneys as part of the protocol. This study provides useful information to consider when developing monitoring and treatment guidelines for kidney oxygenation in the NICU.

## Data Availability

Data is available for sharing upon reasonable requests to the corresponding author and completion of appropriate data sharing agreements with the University of Wisconsin-Madison.
